# Construction Notes

**Published:** 1896-04-25

**Authors:** 


### CONSTRUCTION NOTES.

**Convalescent Home at Llanfairfechan, North Wales.**

This building, the cost of which is being wholly defrayed and endowed by Messrs. Robert, Arthur, and James Heath, the proprietors of the Biddulph Valley Coal and Ironworks, near Stoke-on-Trent, Staffordshire, is being erected for the benefit of the workmen of North Staffordshire. The site is in the Penmaenmair Road, a sheltered spot under the range in hills at Llanfairfechan. The building comprises a three-storeyed block, containing day-rooms and dormitories for fifty beds and matron's rooms, and a basement floor, containing dining-hall, billiard-room, kitchens, &c. The building will be served and heated by hot water throughout and lighted of electricity. A laundry and cottage is also being provided. The external walls are of local stone with stone dressings, and lined with bricks. Mr. Thomas Bower, of Nantwich, Cheshire, is the architect, and Mr. John Gallimore, of Newcastle, Staffordshire, the contractor.

